# Equivalence of care, confidentiality, and professional independence must underpin the hospital care of individuals experiencing incarceration

**DOI:** 10.1186/s12910-023-00891-3

**Published:** 2023-02-21

**Authors:** Markus Eichelberger, Maria M. Wertli, Nguyen Toan Tran

**Affiliations:** 1 Department of General Internal Medicine, Bern University Hospital, Inselspital, University of Bern, Bern, Switzerland; 2 Faculty of Medicine, University of Geneva, Geneva, Switzerland; 3 Australian Centre for Public and Population Health Research, Faculty of Health, University of Technology Sydney, Sydney, NSW Australia

**Keywords:** Incarceration, Human rights, In-patient care, Hospital care, Equivalence of care, Confidentiality, Professional independence

## Abstract

We present the reflections of three clinical practitioners on ethical considerations when caring for individuals experiencing incarceration needing in-patient hospital services. We examine the challenges and critical importance of adhering to core principles of medical ethics in such settings. These principles encompass access to a physician, equivalence of care, patient’s consent and confidentiality, preventive healthcare, humanitarian assistance, professional independence, and professional competence. We strongly believe that detained persons have a right to access healthcare services that are equivalent to those available in the general population, including in-patient services. All the other established standards to uphold the health and dignity of people experiencing incarceration should also apply to in-patient care, whether this takes place outside or inside the prison boundaries. Our reflection focuses on the principles of confidentiality, professional independence, and equivalence of care. We argue that the respect for these three principles, although they present specific implementation challenges, is foundational for implementing the other principles. Critically important are respect for the distinct roles and responsibilities of healthcare and security staff as well as transparent and non-hierarchical dialogue between them to ensure optimal health outcomes and functioning of hospital wards while balancing the ongoing tensions between care and control.

## Background

Individuals in jail and prison—more than 11 million at any given time worldwide [1]—experience poorer mental and physical health than people in the community [2]. The United Nations Nelson Mandela Rules enshrine their right to “enjoy the same standards of healthcare that are available in the community” and “have access to necessary healthcare services free of charge” [3]. The utilization rate of such services—where available and accessible—is higher than that in the general population due to the specific health needs of the population in jails and prisons. For example, in Canada, the utilization rate among individuals experiencing incarceration compared to that in the community at large is approximately five times higher for outpatient care, double for medical-surgical hospitalization, and twenty times higher for psychiatric hospitalization [4].

The delivery of hospital care services differs between and within countries. It can occur outside or inside the boundaries of carceral facilities or as a combination of off-site and on-site services [5]. On-site hospital care could be viewed as more secure and economical from the State perspective [6]. However, there is controversy inherent to such an approach, especially if healthcare professionals are prison employees—the primary interests of patients could be supplanted by healthcare staff’s allegiance to their employer [7].

This paper draws from the authors’ clinical experience in managing university-based hospital wards dedicated to individuals experiencing incarceration, where the criminal justice authorities manage security and carceral issues but have no role in providing healthcare services. Therefore, all three authors are clinicians with no hierarchical affiliation with the criminal justice system. We aim to offer a reflection on the importance and challenges of respecting core principles of medical ethics when organizing and offering off-site or on-site hospital services that best address the in-patient needs of individuals experiencing incarceration.

## Principles of care

The organization and practice of prison medicine, including hospital care, aim to safeguard the health and promote the well-being of individuals experiencing incarceration. Caring for people involved in the criminal justice system must adhere to medical ethics guidelines for prison medicine as formulated in various resolutions, declarations, and recommendations recognized at the international level, national level, or both. These instruments take aim at protecting the rights and dignity of detained individuals. Supranational instruments include the United Nations Nelson Mandela Rules and the guidelines of the European Committee for the Prevention of Torture and Inhuman or Degrading Treatment or Punishment (CPT) [3, 8].

The CPT outlines seven considerations to uphold the health and dignity of people experiencing incarceration: access to a doctor, equivalence of care, patient consent and confidentiality, preventive healthcare, humanitarian assistance, professional independence, and professional competence. Guidance from national medical institutions should align with these standards and be followed by all healthcare professionals attending to individuals experiencing incarceration. For example, the guidelines of the Swiss Medical Sciences Association on health in prison are integrated into the national medical code of conduct. They must be followed by doctors working in carceral settings in Switzerland [9].

Although all seven CPT standards are critical, our discussion will focus on the principles of confidentiality, professional independence, and equivalence of care as they present specific implementation challenges in hospital settings. Our experience has shown that respecting these three principles is foundational for implementing the other principles.

### Medical confidentiality

Medical confidentiality should be respected in accordance with the legal dispositions that apply to people in the community. For example, history-taking and physical examination must take place out of sight and hearing of others, and medical information cannot be shared with the authorities or other third parties without the patient’s informed consent. Keeping patient privacy and confidentiality vis-à-vis security officers and prison authorities is essential to establish and maintain trust between patients and healthcare professionals. However, in a hospital environment, the respect of medical confidentiality is regularly put to the test as medical staff may need to work closely with security personnel, especially when attending to patients with behavior risks. In such cases, security guards can be asked to provide protection against possible physical aggression and, in doing so, may hear the exchange of medical information. Asking the presence of another medical staff as a direct chaperon could help keep security guards at a reasonable distance from the patient-provider interaction and allow to preserve privacy and confidentiality. Therefore, it is critical to regularly remind all healthcare and non-healthcare professionals interacting with this patient population that they are bound by professional secrecy. Put differently, professionals of all involved sectors, including healthcare, security, cleaning, and transportation, among others, are obliged to maintain silence in respect of confidential information they may hear in the course of their duty. This applies not only to the staff of the secure hospital ward but also to all hospital departments where patients go for additional investigations and interventions.

### Professional independence

One of the main challenges we have been facing as healthcare staff caring for in-patients involved in the criminal justice system is to gain and protect their trust. This population is generally suspicious of the criminal justice system and often convinced that healthcare professionals report to the prison authorities—a perception of double loyalty. Double loyalty means that healthcare staff are responsible for the health and well-being of the patient on the one hand (care) and would also have to answer to prison and judicial authorities on the other (control) [10]. Such a situation is a source of ethical, political, and inter-professional tension with potentially deleterious consequences for patient-provider relationships and confidence. Put differently, as soon as individuals experiencing incarceration require care, they are patients and healthcare professionals should unequivocally respond to their needs.

To minimize the risk of double loyalty, we strongly believe that healthcare professionals must be hierarchically independent of the prison administration, even if hospital care is provided within the boundaries of the carceral facility. This means that any hierarchical affiliation or relationship with the prison and judicial authorities must be avoided. Such hierarchical separation of healthcare staff from the authorities must be defended at the coordination level between the healthcare sector and the judiciary. We have found it helpful to reiterate this hierarchical separation with patients to gain and maintain their trust.

The management of the costs of medical care is handled by the prison and criminal justice authorities responsible for enforcing the sentence. This constitutes another potential friction point testing healthcare professionals’ independence. Hospital staff are pressured in their medical decisions to contain costs by limiting diagnostic, treatment, and care options, leading to sub-standard medical care and situations in which the interests of the healthcare team are at odds with those of the prison and judicial authorities. We firmly believe that clinical decisions must be based exclusively on medical criteria, and medical interventions that are not in the interest of the patient should be prohibited. Limiting healthcare interventions due to financial restrictions violates the principle of equivalence of care and aggravates the conflict of double loyalty.

Conflating our duty as caregivers with the duty of experts offers another example of how we experience encroachment on our professional independence. Prison authorities or representatives of the criminal justice system ask medical professionals for their opinion regarding patients’ condition and ability to undertake certain actions or measures after their release from hospital. For example, physicians may be asked to certify that individuals experiencing incarceration can fly to be deported back to the country of origin or continuing the execution of the sentence in prison or the execution of disciplinary measures in solitary confinement cells. We are of the strong opinion that such determination does not fall under the role and responsibility of the caregiver or treating physician—this is the duty of the medical expert, a professional involved specifically in the legal process. Simply put, caregiving and providing medical legal expertise are two conflictual activities that cannot be executed by the same professional; a physician cannot act as medical expert and treating physician at the same time. To safeguard trust between patients and healthcare professionals, we urge caregivers to invoke their primary therapeutic role and non-expert role as well as total independence from the judicial authorities. Therefore, caregivers must defend the hospital ward as a therapeutic and healing space where no such expertise can be carried out.

### Equivalence of care

Respect for the principle of equivalence means that the same preventive, diagnostic, therapeutic, care, and support interventions available to the general population are also available to individuals experiencing incarceration. Violations of this principle are often the result of political and economic pressure to control costs as many detained individuals, particularly those of foreign nationality, do not have healthcare insurance. Their medical costs must be covered by the relevant authorities, which may restrict medical interventions only to urgent and live-saving ones.

Equivalence of care also dictates that patients should be referred and have access to hospital services with competent professionals and adequate equipment that best meet their medical and mental health needs. The lack of appropriate referral infrastructures catering to individuals experiencing incarceration could result in patients receiving sub-optimal care compared to the general population. As a general approach, patients with acute psychiatric decompensation must be hospitalized in a secure psychiatric ward; in case of physical comorbidities, they are cared for by health staff specializing in internal medicine or family medicine. Conversely, individuals with surgical or internal medicine conditions requiring inpatient care must be hospitalized in a medical ward; in case of mental comorbidities, they are cared for by mental health specialists. Hospitalization in specialized services must be guaranteed, such as for patients requiring intensive care, obstetric care, pediatric care for those under 16 years of age, psycho-geriatric care, or palliative care. Although hospitalization in these specialized wards may be perceived as an additional security risk, we firmly believe that healthcare professionals must demand unimpeded access to unshackled patients. The latter points to the essence of equivalence of care: individuals experiencing incarceration have the right to enjoy high-quality and person-centered care as does the general population. Such quality of care is underpinned, among others, by respect, dignity, privacy and confidentiality, safety, and informed decision-making, which health professionals should aim to offer to all individuals without discrimination [11].

### Further reflections

Providing care in a hospital environment can be more complex and challenging for healthcare professionals due to security regulations. We believe that respect for the distinct roles and responsibilities of healthcare and security staff as well as transparent and non-hierarchical dialogue between them are essential for the optimal health outcomes and functioning of the hospital ward as well for balancing the tensions between “care and control” [10, 12]. The rules and living conditions in hospital settings are managed by the carceral authorities and are often more restrictive than in prison (e.g., increased use of shackles and restrictions on walking, smoking, telephone, or visits). This is due to the perception of increased security threats, such as escaping and hostage-taking, especially as patients often require investigations that are available only in other non-secured hospital services. Preparing patients for their hospitalization with clear information on rules and living conditions can optimize patient compliance and spare resources.

Although medical ethics guidelines are undisputed, our experience has shown that implementing them in hospital settings is challenging. Therefore, on-site and off-site in-patient facilities for individuals experiencing incarceration should be regularly monitored and even accredited by independent expert groups [13].

## Conclusion

Healthcare professionals attending to individuals experiencing incarceration work in criminal justice systems that are often used as instruments of oppression, marginalization, and racial discrimination [14]. As such, health professionals must strive to ensure due respect for human rights and ethical principles, as these form the foundation of quality of care. We are convinced that the respect for the principles of equivalence of care, confidentiality, and professional independence by healthcare staff as well as prison and criminal justice authorities is essential to uphold patients’ dignity and ensure equitable treatment. Ultimately, whenever caregivers are torn between their professional and ethical standards and demands from authorities that do not align with such standards, we call upon them to rely on and act in accordance with their highest professional and ethical principles and refuse to cooperate in actions that would contradict such principles.

## Data Availability

Not applicable.

## Abbreviations

CPT: European Committee for the Prevention of Torture and Inhuman or Degrading Treatment or Punishment.
